# Comparison of Two Methods Forecasting Binding Rate of Plasma Protein

**DOI:** 10.1155/2014/957154

**Published:** 2014-08-04

**Authors:** Liu Hongjiu, Hu Yanrong

**Affiliations:** Changshu Institute of Technology, Changshu 215500, China

## Abstract

By introducing the descriptors calculated from the molecular structure, the binding rates of plasma protein (BRPP) with seventy diverse drugs are modeled by a quantitative structure-activity relationship (QSAR) technique. Two algorithms, heuristic algorithm (HA) and support vector machine (SVM), are used to establish linear and nonlinear models to forecast BRPP. Empirical analysis shows that there are good performances for HA and SVM with cross-validation correlation coefficients *R*_cv_^2^ of 0.80 and 0.83. Comparing HA with SVM, it was found that SVM has more stability and more robustness to forecast BRPP.

## 1. Introduction

Pharmacokinetic (PK) often uses mathematical models and equations to study quantitative change law of medicines with time [1, 2]. PK is divided into several areas including the extent and rate of absorption, distribution, metabolism, and excretion [3]. It is mainly used to build mathematical expressions to monitor individual in vivo dose or drug regimen with time and work out PK parameters, make out, and adjust individual regimen to guarantee effectiveness and safety of treatment by applying PK model, expression, and parameters [4, 5]. After a drug is absorbed into vein, most of it is bound with plasma protein. Combining percentage of a drug for therapeutic dose and plasma protein is called binding rate of plasma protein (BRPP) [6]. In this paper, BRPP is stable because it is a measured value for normal people in normal dose. Free drug can diffuse to organisms by lipid membrane. And it can be filtered by tubules or metabolized by liver [7]. Consequently, combination of drug and protein can have evident effect on process of drug distribution and elimination and decrease drug potency at the target site. Some studies indicate that pharmacodynamic and pharmacokinetic is mainly influenced by its binding protein, so does bioavailability [7–10]. The higher BRPP is, the longer its half-life is. Among R&D projects, about percent forty candidate compounds fell into disuse because of poor PK parameters, such as slow absorbing speed, low bioavailability, high BRPP, quick metabolization leading to short duration of drug action, and metabolites with toxicity and slow excretion leading to accumulated toxicity, in a body [11]. These reasons make in vitro activity of the compounds to lose the developing values of clinical drugs [12]. Therefore, for feasible drug design, we must consider characteristics of pharmacodynamic and pharmacokinetic to achieve the best balance between them. It is an important content of drug design for quantitative structure pharmacokinetic relationship (QSPKR) [13, 14] and quantitative structure-activity relationship (QSAR) [15, 16]. At the same time, they are also successfully used to forecast characteristics of drugs such as drug metabolism, toxicity, and actual bioavailability. Computer-aided drug design (CADD) is becoming an important research field of new drug development [17], which can apply known knowledge of drug molecules and biological targets to find and design new kinds of drug molecules by theoretical simulation and calculation [18]. At present, it is a very active area to study PK models in pharmaceutical industry. Because drug BRPP is influenced by many factors, causality and mechanism are not clear and distinct between molecular structures of drugs. As far as the present scientific level is concerned, there are still many difficulties to clarify relations between them according to basic principles. Classical forecasting methods (e.g., multiple linear regression) face more and more dilemmas. However, artificial intelligence methods provide stronger tools to analyze existed PK data and construct QSPKR between BRPP and molecular structure variables of a drug. In particular, the results of practical application in other fields indicate that the performance of support vector machine (SVM) has superiority over ANN and can overcome the problems of overfitting and local minimization of traditional neural networks excellently.

In order to find a new method to construct a PK model of BRPP, we establish QSAR models by Heuristic algorithm (HA) and SVM with BRPP of seventy drugs and test forecasting performance and stability of a SVM model.

The remainder of the paper is organized as follows. Principles of research methods are introduced in Section 2. Empirical study is presented in Section 3. Finally, conclusive results are drawn in Section 4.

## 2. Data Resource and Methodology

### 2.1. Data Resource and Structure Parameters

All experimental data of seventy drugs and their BRPPs resource are from reference [19]. Models are constructed by training set consisting of fifty-six drugs chosen randomly. Data of the remaining fourteen drugs as test set are used to examine stability and forecast performance of the two models. All compounds are initially optimized by molecular mechanics method (MM+) in program Hyperchem 4.0. Then, they are geometrically optimized further by semiempirical method (AM1). Optimized molecular structure is calculated in MOPAC 6.0, and then the results are transferred into CODESSA program to calculate five kinds of descriptors (independent variables): composition descriptor, topological descriptor, geometric descriptor, electrostatic descriptor, and quantum chemical descriptor.

### 2.2. Heuristic Algorithm

HA can entirely search for a great quantity of molecular descriptors in software CODESSA and establish optimal linear regression equation [20]. HA has to control collinearity of molecular descriptors [21]. For example, if correlation coefficient of any two descriptors is more than 0.8, they will not be involved in the same model simultaneously. The optimal model is built by rapid filter and selection of HA to descriptors, while it is not done by considering all possible combination of descriptors. HA takes pretreatment way to eliminate some descriptors according to four rules: (1) the descriptors not owned by each compound; (2) descriptors with smaller changes of values for all compounds; (3) descriptors with *F* test value less than 1.0 in an equation; (4) descriptors with *t* test value less than a specific value [16]. Heuristic regression method (HRM) sequences molecular descriptors as descending order of correlation coefficients of a model. Every time, the descriptor with the biggest correlation coefficient is introduced among the remaining descriptors, which takes turn until the end. Performance of a model depends on multiple correlation coefficient (*R* ^2^), *F* test value (*F*), standard deviation (*s*), and so forth [22]. Stability of a model is tested by correlation coefficient *R*_cv_^2^ of cross-validation of leave-one-out (LOO) [23]. Briefly, eliminate a sample in data set and forecast the eliminated sample by building a new model with the same descriptors, take turns until every sample in data set is eliminated and forecasted once, and calculate correlation coefficient between a forecasted value and an observed value. Generally, speed and quality of HRM are higher than others, which makes it become the first choice in practice [24].

In this paper, errors of heuristic regression results are denoted by root mean square (RMS), and the equation is as follows:

(1) $$\begin{array}{c} \text{RMS}=\sqrt{\frac{\sum_{i=1}^{n_{s}}{\left(y_{k}-{\hat{y}}_{k}\right)}^{2}}{n_{c}}}, \end{array}$$

where *y*_*k*_ is target value, ${\hat{y}}_{k}$ is an observed value, *n*_*c*_ is the quantity of compounds, and *c* denotes a compound.

### 2.3. Support Vector Machine

Principle of SVM is that maps input vector *x* into high-dimensional feature space by scheduled nonlinear mapping and then constructs optimal hyperplane in the high-dimensional space [25]. Thus, the problem is transformed into quadratic programming. No matter what target function or classification function it is, they both involve the inner product in quadratic programming. If a kernel function is used, it can avoid complex calculations in high-dimensional space and realize the inner calculations by an original space function. Consequently, selecting appropriate inner product *K*(*x*_*i*_, *y*_*i*_) can realize linear calculation of a nonlinear transformation, while it does not increase calculating complexity [26]. Support vector machine regression (SVRM) maps a variable *x* into high-dimensional feature space by a nonlinear constructor Φ, and the regression is done in the space [27].

Assume the given input sample *x* is a *n*-dimension vector, *k* samples and their output value *y* are denoted as follows:

(2) $$\begin{array}{c} \left(x_{1},y_{1}\right),\ldots ,\left(x_{k},y_{k}\right)\in R^{n}\times R. \end{array}$$

Regression analysis is also called function estimation, which is a statistical process for estimating the relationships among variables. For a given sample set {(*x*_*i*_, *y*_*i*_), *i* = 1,…, *k*}, where *x*_*i*_ is the *i* independent factor (descriptor) and *y*_*i*_ is the *i* dependent factor (BRPP). A regression model relates *y* to a function of *x*, *y* = *f*(*x*). If the function *f*(*x*) is linear, the regression is called as linear regression, otherwise called as nonlinear regression [28]. There is only one kind of sample points for SVMR, namely, optimal hyperplane which makes the total deviation minimized between all sample points and the hyperplane. Thus, sample points are between two borders. If insensitive function *ε* is taken as an error function, the problem of how to find the optimal regression hyperplane is transformed to solve quadratic convex programming when the distances of all sample points to the quested hyperplane are not more than *ε* [25]. Namely,

(3) min⁡ 12||w||2,S.T. yi−(w·xi)−b≤ε,   (w·xi)+b−yi≤ε.

When distances of several sample points to the hyperplane are more than *ε*, deviation of insensitive function *ε* is equivalently the introduced slack variable *ξ*_*i*_ of SVM clustering. Introducing fault-tolerant penalty function *C*, the problem of quadratic convex programming to find the optimal regression hyperplane can be transformed as follows:

(4) min⁡ 12||w||2+C∑i(ξi+ξi∗),S.T.   yi−(w·xi)−b≤ε+ξi,   (w·xi)+b−yi≤ε+ξi∗,   ξi,ξi∗≥0.

Then, linear regression function of the optimal hyperplane is

(5) $$\begin{array}{c} f\left(x\right)=\left(w\cdot x\right)+b=\underset{\text{S}.\text{V}.}{\sum }\left(a_{i}-a_{i}^{*}\right)\left(x\cdot x_{i}\right)+b. \end{array}$$

*a*_*i*_, *a*_i_*, and *b* can be calculated through constraints; S.V. denotes support vector. In order to determine parameters of the optimal hyperplane, the above solving process can be realized by MATLAB program. Last results indicate that the optimal regression hyperplane is only determined by sample points. If points *x* and *x*_*i*_ in sample space are replaced by mapped image point *ψ*(*x*) and *ψ*(*x*_*i*_) with a kernel function; let *K*(*x*, *x*_*i*_) = (*ψ*(*x*) · *ψ*(*x*_*i*_)); *L* denotes number of points [29]; then,

(6) $$\begin{array}{c} f\left(x\right)=\left(w\cdot \psi \left(x\right)\right)+b=\overset{L}{\underset{i=1}{\sum }}\left(a_{i}-a_{i}^{*}\right)K\left(x,x_{i}\right)+b. \end{array}$$

## 3. Empirical Analysis

### 3.1. HA Model

**Figure 1:**
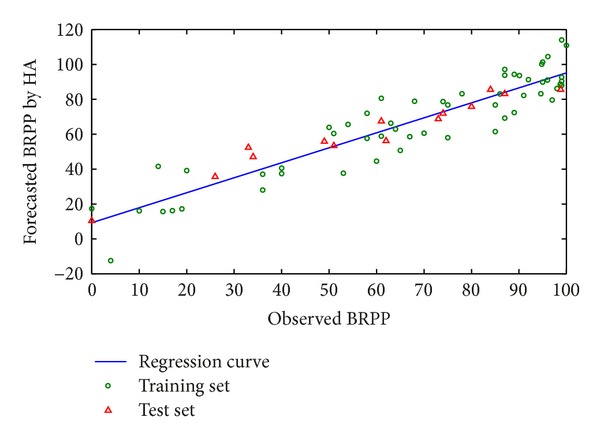
Forecasted BRPP and observed BRPP based on HA.

Each molecule can be worked out to five hundred to six hundred descriptors by using CODESSA, including composition, topological, geometric, electrostatic, and quantum chemical descriptors. Composition descriptor reflects the composition information of a molecule, including quantity of atoms, atomic bonds, atomic rings, and molecular weight. Topological descriptor indicates connecting information of atoms in a molecule, including Wiener index, Randic index, and Kier-Hall index. Geometric descriptor reveals size and shape of a molecule, including inertia moment, molecular cubage, and surface area. Electrostatic descriptor displays distribution information of electric charges in a molecule, including maximum and minimum partial charges, polarity, and charged partial surface area (CPSA). Quantum chemical descriptor discloses electric charge distribution in a molecule and energy information of molecular orbit, including reaction index, dipole moment, energy of lowest unoccupied molecular orbital (LUMO), and highest occupied molecular orbital (HOMO), which has an important effect on molecular reaction, electrostatic interaction between molecules, and interaction between molecular orbits. By HM filtering, six parameters are introduced to the model. Their interrelations and forecasting results are seen in Tables 1 and 2. In HA model, correlation coefficient *R* ^2^ = 0.85, test value *F* = 63.64, error RMS = 12.24, and correlation coefficient of cross-validation *R*_cv_^2^ = 0.80 (see Figure 1 and Table 3).

There are six descriptors in HA linear model. WPSA-3 weighted PPSA (Zefirov's PC), HASA-1/TMSA (Zefirov's PC), and PNSA-2 total charge weighted PNSA are electrostatic descriptors. ALFA polarizability (DIP), Tot point-charge compd. of the molecular dipole and final heat of formation are quantum chemical descriptors. *α*-polarizability is molecular polarizability which reflects molecular cubage and interaction between agent and molecule. Polarizability scale is closely related to hydrophobicity and electrophilicity. In the model, only the signal of *α*-polarizability parameter is positive, which indicates that polarizability has a positive effect on bond of drug and plasma protein. Hydrophobicity influences combination of drug and plasma protein directly. Because protein consists of polypeptides with electric charge, the stronger electrophilicity is, the easier binding with plasma protein is. Final heat of formation (FHF) is relative to molecular stability, which expresses molecular reaction ability. Change of FHF influences molecular structure and function, while it does the combination of drug molecule and plasma protein. WPSA-3 weighted PPSA is partial positive surface charge. PNSA-2 total charge weighted PNSA is the weights of total charges and determined by surface area and functional gene of molecule, which reflects interactions between polarmolecules. On the surface of plasma protein, there are enzymes with specific function gene. At the same time, there also exist a series of receptors. As the ligand, drug is bound with receptors on the surface of plasma protein. Consequently, change of function gene on the surface of drug molecule can have a significant effect on BRPP. HASA-1/TMSA is a ratio of surface area of hydrogen bond receptor to total surface area of molecule, which is a weight area of surface charge of hydrogen bond donor atoms. According to the above explanation, dipole moment between molecules and the surface of hydrogen-bonding acceptor and molecular surface are both main influencing factors of bond of drug and plasma protein. Tot point-charge compd. of the molecular dipole is the contribution of point charge to molecular dipole moment.

### 3.2. SVM Model

In order to compare performance of SVM with that of HA model, we choose the same test set, training set, descriptors with HA model. In SVM model, it is very crucial to choose kernel function. There are four kinds of kernel functions including linear, polynomial, Gaussian, and sigmoid. When size and dimension of samples are small, the four kernel functions can show better performance. On the contrary, Gaussian kernel is a better choice [30], which is most commonly used in SVMR; namely,

(7) $$\begin{array}{c} F\left(u,v\right)=\exp\left(-\gamma^{*}{\left|u-v\right|}^{2}\right), \end{array}$$

where *γ* is a constant, *u* and *v* are two independent variables. *γ* controls generalization ability of SVM by adjusting the shape of Gaussian function. Because size and dimension of samples are big in our study, Gaussian kernel function is a preferred choice. The forecasting results are seen in Table 2. After adjusting *γ*, *ε*, and *C* simultaneously, we can get three useful results.

**Figure 2:**
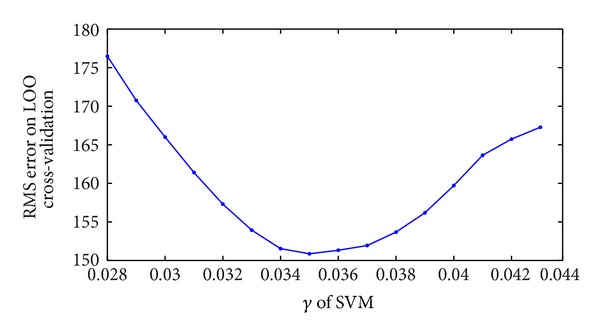
Relation of *γ* and RMS error on LOO cross-validation.

**Figure 3:**
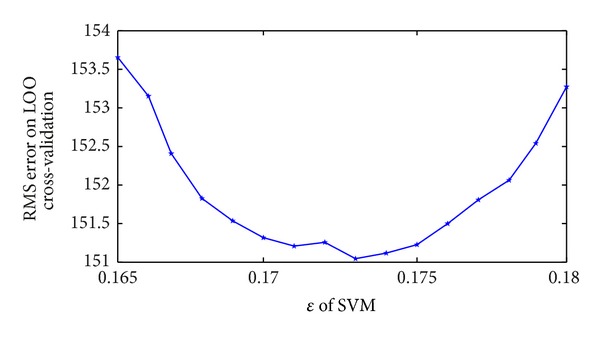
Relation of *ε* and RMS error on LOO cross-validation.

Firstly, seeing Figure 2, the error is minimal when *γ* is 0.035. Optimal value of *ε* depends on data type while it also considers support vectors. Because insensitive function *ε* can control border of all training set, it is very important for SVM to choose *ε*. Secondly, relation between *ε* and errors is seen in Figure 3. When *ε* = 0.173, the error is the least.

**Figure 4:**
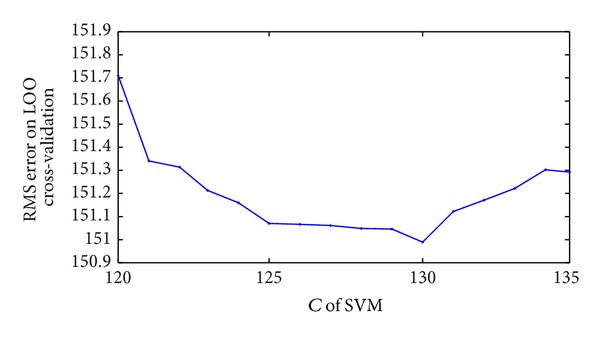
Relation of *C* and RMS error on LOO cross-validation.

Thirdly, another important parameter *C* is used to measure the training error between maximal and minimal hyperplane. If *C* is too small and training is not enough, it is very difficult to arrive to the optimal. On the contrary, overfitting phenomenon will happen. Relation between *C* and errors is seen in Figure 4. When *C* is equal to 130, the error is the least.

**Figure 5:**
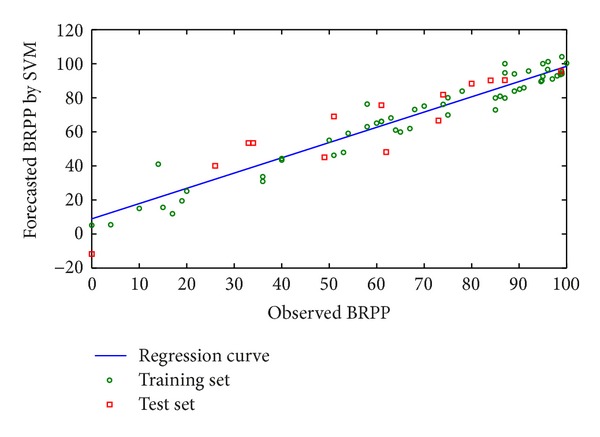
Forecasted BRPP and observed BRPP of HA.

According to the above training results, when the optimal parameters *γ*, *ε*, and *C* are equal to 0.035, 0.173, and 130 respectively, forecasting ability of the model is the most robust and stable. In Figure 5, RMS is 11.40. For training and test set, *R* ^2^ is 0.97 and 0.92, respectively. Total *R*_cv_^2^ is 0.83.

Comparing HA with SVM, it is found that their correlation coefficient square (*R* ^2^) is 0.80 and 0.83, respectively, after cross-validation, RMS is 12.24 and 11.40, respectively. Higher *R* ^2^ value and lower RMS value indicate a better predictability of the dependent variable from the independent variables [31]. Therefore, a conclusion can be drawn that SVM model has better stability and more robust forecasting ability for BRPP than HA model, which is a good tool to construct PK a model.

## 4. Conclusion

In this paper, we construct HA and SVM model to forecast BRPP, respectively. By calculating descriptors of molecular structure, we found that it is satisfactory for forecasting results of nonlinear QSAR model based on SVM and linear QSAR model based on HA. By comparison of two methods, nonlinear model based on SVM is more stable and more robust to forecast BRPP than linear model based on HA. Therefore, SVM model is a more effective tool to study QSAR and BRPP of a drug.

However, because the comparison is primarily based on the analysis of one real dataset, our research has certain limitations. The conclusions need research supports of more datasets. SVM performance of predicting BRPP should be studied and discussed further by more datasets in the future.

## Figures and Tables

**Table 1:** Correlation matrix of the six descriptors^a^.

Descriptor	1	2	3	4	5	6
1	1.000	0.177	0.776	0.039	−0.190	−0.559
2		1.000	0.266	0.269	−0.104	−0.255
3			1.000	0.129	−0.541	−0.592
4				1.000	−0.192	−0.497
5					1.000	0.325
6						1.000

^a^1: ALFA polarizability (DIP), 2: WPSA-3 weighted PPSA (Zefirov's PC), 3: HASA-1/TMSA (Zefirov's PC), 4: Tot point-charge compd. of the molecular dipole, 5: PNSA-2 total charge weighted PNSA, and 6: final heat of formation.

**Table 2:** Experimental and calculated BRPP based on HA and SVM.

No.	Compd.	BRPP/(%)	HA	Residual	SVM	Residual
1^a^	Acebutolol	26.0	35.9	9.9	40.0	14.0
2	Alprenolol	85.0	61.5	−23.5	72.9	−12.1
3	Amantadine	67.0	58.6	−8.4	61.9	−5.1
4	Amiodarone	100.0	110.9	10.9	100.3	0.3
5	Amitriptyline	94.8	100.1	5.3	90.1	−4.7
6^a^	Aspirin	49.0	56.1	7.1	45.0	−4.0
7	Betamethasone	64.0	62.9	−1.1	61.0	−3.0
8	Bumetanide	99.0	92.6	−6.4	94.0	−5.0
9	Caffeine	36.0	28.0	−8.0	30.9	−5.1
10	Cefalexin	14.0	41.6	27.6	41.0	27.0
11^a^	Chloroquine	61.0	67.7	6.7	75.6	14.6
12	Chlorthalidone	75.0	76.8	1.8	80.0	5.0
13	Cimetidine	19.0	17.2	−1.8	19.4	0.4
14	Ciprofloxacin	40.0	40.6	0.6	43.4	3.4
15	Diphenhydramine	78.0	83.2	5.2	83.9	5.9
16^a^	Furosemide	98.8	85.8	−13.0	95.5	−3.3
17	Glibenclamide	99.0	114.0	15.0	95.1	−3.9
18	Haloperidol	92.0	91.3	−0.7	95.7	3.7
19	Lidocaine	70.0	60.6	−9.4	75.1	5.1
20	Methadone	89.0	94.3	5.3	94.0	5.0
21^a^	Methotrexate	34.0	47.3	13.3	53.4	19.4
22	Metoclopramide	40.0	37.4	−2.6	44.3	4.3
23	Metronidazole	10.0	16.1	6.1	15.0	5.0
24	Nifedipine	96.0	91.0	−5.0	96.6	0.6
25	Phenobarbital	51.0	60.4	9.4	46.2	−4.8
26^a^	Pindoioi	51.0	53.6	2.6	69.0	18.0
27	Prednisone	75.0	58.0	−17.0	69.9	−5.1
28	Quinidine	87.0	93.8	6.8	100.0	13.0
29	Ranitidine	15.0	15.7	0.7	15.6	0.6
30	Sulfadiazine	54.0	65.6	11.6	59.1	5.1
31^a^	Sulfamethoxazole	62.0	56.4	−5.6	48.1	−13.9
32	Terbutaline	20.0	39.2	19.2	25.1	5.1
33	Timolol	60.0	44.6	−15.4	65.1	5.1
34	Triamterene	61.0	58.9	−2.1	66.1	5.1
35	Amikacin	4.0	−12.4	−16.4	5.4	1.4
36^a^	Carbamazepine	74.0	72.2	−1.8	81.8	7.8
37	Carbenicillin	50.0	63.9	13.9	55.0	5.0
38	Cefamandole	74.0	78.7	4.7	76.1	2.1
39	Cefazolin	89.0	72.4	−16.6	83.9	−5.1
40	Cefotaxime	36.0	37.1	1.1	33.6	−2.4
41^a^	Cefuroxime	33.0	52.6	19.6	53.4	20.4
42	Chloramphenicol	53.0	37.7	−15.3	47.9	−5.1
43	Chlorothiazide	94.6	83.2	−11.4	89.5	−5.1
44	Clonazepam	86.0	83.1	−2.9	80.9	−5.1
45	Cocaine	91.0	82.2	−8.8	85.9	−5.1
46^a^	Dapsone	73.0	69.0	−4.0	66.6	−6.4
47	Dexamethasone	68.0	78.9	10.9	73.1	5.1
48	Diazepam	98.7	88.7	−10.0	93.6	−5.1
49	Ethinylestradiol	98.0	86.2	−11.8	92.9	−5.1
50	Famotidine	17.0	16.2	−0.8	11.9	−5.1
51^a^	Fentanyl	84.0	85.8	1.8	90.2	6.2
52	Flecainide	61.0	80.6	19.6	66.0	5.0
53	Hydrochlorothiazide	58.0	57.6	−0.4	63.0	5.0
54	Imipramine	90.1	93.6	3.5	85.0	−5.1
55	Isoniazid	0.0	17.4	17.4	5.1	5.1
56^a^	Isosorbide-5-mononitrate	0.0	10.7	10.7	−11.8	−11.8
57	Ketoconazole	99.0	90.4	−8.6	104.1	5.1
58	Lovastatin	95.0	101.3	6.3	100.0	5.0
59	Mexiletine	63.0	66.3	3.3	68.1	5.1
60	Nitrazepam	87.0	97.1	10.1	94.6	7.6
61^a^	Norethisterone	80.0	76.1	−3.9	88.3	8.3
62	Omeprazole	95.0	89.9	−5.1	92.6	−2.4
63	Pethidine	58.0	72.0	14.0	76.3	18.3
64	Phenylbutazone	96.1	104.5	8.4	101.2	5.1
65	Propafenone	97.0	79.6	−17.4	91.0	−6.0
66^a^	Propranolol	87.0	83.4	−3.6	90.3	3.3
67	Pyrimethamine	87.0	69.2	−17.8	79.8	−7.2
68	Thiopental	85.0	76.8	−8.2	79.9	−5.1
69	Ticarcillin	65.0	50.7	−14.3	59.9	−5.1
70	Warfarin	99.0	88.0	−11.0	94.0	−5.0

^a^test set.

**Table 3:** Correlation coefficient of six-descriptor in HA model.

Descriptor	Coefficient	*t*-test
ALFA polarizability (DIP)	0.653 ± 0.056	11.755
^ a^WPSA-3 Weighted PPSA (Zefirov's PC)	−10.969 ± 0.860	−12.756
^ a^HASA-1/TMSA (Zefirov's PC)	−73.908 ± 14.802	−4.993
Tot point-charge compd. of the molecular dipole	−7.799 ± 0.918	−8.495
^ a^PNSA-2 Total charge weighted PNSA	−0.036 ± 0.005	−7.757
Final heat of formation	−0.059 ± 0.016	−3.596
R^2^ = 0.85, *F* = 63.64, RMS = 12.24, *R*_cv_^2^ = 0.80		

^a^TMSA: total molecular surface area; PNSA: partial negative surface area; PPSA: partial positive surface area.
